# Mental Health Needs of Families of Patients in Intensive Care Units and the Role of Mobile Health: Survey Study

**DOI:** 10.2196/75461

**Published:** 2026-03-20

**Authors:** Anthony Faiola, Saira Soroya, Zhonglin Hao, Reinhold Munker

**Affiliations:** 1 Department of Population Health College of Nursing University of Cincinnati Cincinnati, OH United States; 2 College of Information and Library Science University of Southern Connecticut State University Hartford, CT United States; 3 College of Medicine Markey Cancer Center University of Kentucky Lexington, KY United States; 4 Division of Hematology, College of Medicine Markey Cancer Center University of Kentucky Lexington, KY United States

**Keywords:** mental health, intensive care unit, ICU, communication, mobile health, COVID-19, health equity, health technology, digital tools, eHealth

## Abstract

**Background:**

The experiences of patients with COVID-19 and their families manifested the most devastating effects of family separation since the 1918 Spanish influenza pandemic and, with it, a call for solutions to patient isolation and its effect on family mental health.

**Objective:**

This study examined the recent experiences of families of critical care (intensive care unit; ICU) patients related to anxiety and depression (AD), satisfaction with clinician-family communication, and counseling from mental health and social services. This study explored correlations between these factors and family interest in mobile health (mHealth) designed to improve information flow and communication from patient bedside to remote families.

**Methods:**

Using a 36-question quantitative survey, we collected 97 responses over 6 months. We selected participants by using a convenience sampling strategy. To analyze data, we applied descriptive and inferential statistics. Participants represented a spectrum of ages, relationships to patients, and races (n=78, 80% White; n=17, 18% Black; n=2, 2% other races). Approximately 17% (n=16) of the patients were admitted for cancer, 13% (n=13) were admitted for COVID-19, and 21% (n=20) were admitted for other conditions.

**Results:**

The mean score for remote families’ satisfaction with patient health updates from the bedside and mental health services was 2.94 (SD 1.31), whereas that for phone communication was lower on average. The mean scores of family AD levels were elevated, and levels were higher among family members during the ICU stay than after discharge. These findings confirmed evidence of a negative correlation between transportation difficulties and satisfaction with the frequency of information provided (*r*=−0.284; *P*=.005), suggesting that, with the increase in transportation challenges, families become less satisfied with the frequency of patient health information. Family members expressed strong interest in using mHealth information and communication services (mean 8.34, SD 1.98) and having easy access to social workers to manage AD (mean 8.29, SD 2.03). Families experiencing higher levels of anxiety during patients’ ICU stays had significantly greater interest in the use of an mHealth app that would provide direct access to social workers (*r*=0.326; *P*<.001), in using an mHealth videoconferencing app (*r*=0.319; *P*=.002), and in overall mHealth app use (*r*=0.322; *P*<.001).

**Conclusions:**

Family members experienced high levels of AD during patient ICU admission, as well as after discharge even though their mental health challenges were reduced. Families were highly dissatisfied with the frequency of health updates, with lower satisfaction reported among those who faced difficulties arranging transportation or lived further from the hospital. Modest but statistically significant correlations were observed between family members’ reported mental health status during ICU stays and an interest in an mHealth app that could provide access to real-time bedside information, facilitate communication with bedside nurses, and support connections with social workers.

## Introduction

The experiences of patients with COVID-19 and their families in the intensive care unit (ICU) brought to the fore the most devastating effects of family (or caregiver) separation since the 1918 H1N1 Spanish influenza pandemic [1]. Today, even under the best of conditions, ICU patients and their families often experience uncertainty and heightened levels of confusion that inevitably produce increased psychological burden [2]. The ICU is a critical care facility dedicated to patients who need constant specialized bedside care [3], a situation in which the family members of patients develop high levels of anxiety and depression (AD).

Multiple studies show that this is due in part to a lack of timely medical updates and staff communication, especially if family members live in locations far from the point of care [4-6]. In particular, studies demonstrate that family members are more likely to develop anxiety, depression, posttraumatic stress disorder [7-9], and disruptions to family relationships [10,11] because of challenges surrounding less than optimal information flow from bedside to family members [12].

Other key factors impacting the mental health of family members of ICU patients include isolation from the patient [13-20], socioeconomic inequalities [21-23], and increased transportation needs due to interhospital transfers [24-26]. For example, during the COVID-19 pandemic [27], families encountered the suspension of ICU visitation rights and, as a result, experienced a high prevalence and severity of acute stress disorder even 3 months after patient discharge [8]. Researchers have argued that “during times of healthcare crises and the restriction of the physical presence of families, family support is more, not less, important” [28,29]. As a result, there is a need for increased communication between the ICU care team and family members, adopting “family-centered tools” that address restrictions on family access to patient health updates and communication [30-33].

Such tools might include family-centered digital solutions that provide relevant and easily accessible information from the bedside for family members. Research suggests that convenient ways to support families in their understanding of the real-time health status of patients might include mobile apps (mobile health; mHealth). Such interventional support would be specifically designed to safely and securely communicate patient health and wellness information to families, providing them with the greatest benefit to their mental health [34-36].

Researchers further argue that health care providers should explore mHealth as part of standardized care for patient families [37]. To address these challenges, digital communication interventions are one possible solution [38,39] to reduce the psychological burden on family members [40,41] with a loved one in the ICU. In support of this view, it is well documented in prior studies that particular mHealth apps have the potential to significantly reduce AD. For example, in one systematic review and meta-analysis, researchers observed 15 studies that reported on the positive effects of mHealth interventions on AD symptoms in patients with cancer, with the mHealth intervention group demonstrating a statistically significant improvement in AD compared to the control groups. The researchers concluded that their meta-analysis demonstrated the potential of mHealth interventions to significantly improve anxiety, depression, and quality of life in patients with cancer [42].

A second systematic review addressing the effects of mHealth, web-based, or virtual reality platforms on depression, anxiety, and enhancement of psychological well-being among college students found that the vast majority of studies reported that digital interventions were either effective or partially effective in producing beneficial changes in the main psychological outcome among students. The effectiveness of the intervention did not appear to substantially vary by type of digital mental health technology used, indicating that all held the potential for improving mental health on college campuses [43].

Another scoping study of 8 studies demonstrated the positive effect of mHealth to reduce mental health burdens among family members. The results showed decreased depression scores, with strong satisfaction with the accessibility and flexibility of mHealth combined with peer support features [44].

These well-documented studies suggest that mHealth has the potential to support higher patient and family satisfaction and the lowering of family member AD [45]. To this end, we designed this study to provide additional insight into the needs of families of critical care patients. Hence, the broad objective of this study was to assess to what degree increased access to ICU patient health information via mHealth or other forms of digital communication may serve as a strategic intervention to reduce AD among patients’ family members.

We present the current experiences of family members of ICU patients; gauged their interest in alternative communication solutions that would help lower their experiences of AD; and explored any associations among family interests in increased communication, family AD, and family satisfaction with current communication systems within the health care system.

This study was designed to answer the following 5 specific research questions (RQs):

- RQ 1: to what extent are family members satisfied with patient health information communicated by ICU medical staff?
- RQ 2: what is the prevalence of AD among family members during the patients’ stay in the ICU and after discharge?
- RQ 3: is there any relationship between the difficulty in arranging transport to and from the hospital and family members’ level of satisfaction with the frequency of communication (RQ 3a) and AD (RQ 3b)?
- RQ 4: how interested are families in the use of a mobile app that sends patient health updates and allows for communication with nurses, social workers, and/or mental health counselors via SMS text message and/or video about the patients’ health status?
- RQ 5: is there a correlation between family members’ AD and their satisfaction with current communication with the critical care team?

## Methods

### Study Design

We collected quantitative data using a 36-question survey divided into six categories: (1) demographics (multiple choice; questions 1-2); (2) patient and family background, such as location and medical history (multiple choice; questions 6-15); (3) degree of AD and general mental health (sliding scale; questions 16-23); (4) quality and quantity of health care services and communication (sliding scale; questions 24-30); (5) degree of health inequity experienced while in the hospital (sliding scale; questions 31-33); and (6) degree of interest in the use of mHealth to facilitate better communication with the clinical staff (sliding scale; questions 34-36).

The Family Inpatient Communication Survey [46,47] and senior technology acceptance model survey [48], along with other published papers addressing family-centered care of patients [20,49-51], provided guidance in the development of our research instrument. After institutional review board approval, we prepared the questionnaire in the REDCap (Research Electronic Data Capture; Vanderbilt University) survey platform. The overall reliability of the scales used was assessed using the Cronbach α, yielding a coefficient of 0.758, indicating acceptable internal consistency.

All self-reported psychological constructs within the survey, such as anxiety, depression, satisfaction, and mental health, were left to each participant’s interpretation based on their subjective experience of the terms. For example, one question asks the following: “How would you rate your anxiety after your family member was in the ICU?” Next to the question is a sliding scale with no numbers, ranging from “no anxiety” on the left to “highly anxious” on the right. Such terms were not operationalized or linked to existing standardized psychometric tools such as the Hospital Anxiety and Depression Scale or the Beck Anxiety Inventory [52].

### Recruitment

For participant enrollment, we posted our survey aims and invitation with a URL on 16 social networking websites and Facebook community groups related to critical care and family-centered care. We also displayed posters at selected clinical sites on the University of Kentucky medical campus (Lexington, Kentucky) for further recruitment. Individuals were eligible to participate if they were (1) adults (aged ≥18 years) and (2) self-identified family members of critical care patients (in cardiac, pediatric, COVID-19, and cancer ICUs) admitted within the previous 2 years. Data collection lasted 6 months.

### Sample Size

This research project was originally designed as a proof-of-concept study, where a limited sample would be sufficient to achieve our intended goals. Moreover, we experienced extraordinary challenges in obtaining responses. As such, we set the sample size at 100 using a convenience sampling method for participant recruitment. A post hoc sensitivity analysis was performed using G*Power (version 3.1). With a sample size of 100, an α level of .05, and statistical power set at 80%, the analysis demonstrated sufficient capacity to detect a medium effect size of *r*=0.28 (2 tailed). This indicates that the study was appropriately powered to identify moderate or stronger associations, aligning with the objectives of this pilot investigation. We received 97 responses in the end.

### Data Analysis

We analyzed the data using the open-source statistical software Jamovi (version 2.7.9) [53]. Jamovi is a statistical software built on R (R Foundation for Statistical Computing). Standard significance levels were used throughout the analysis, with α=.05 as the threshold for statistical significance unless otherwise specified. To analyze the data, first, we examined the extent and pattern of missingness across variables. Jamovi excludes any row (case) that has missing data for one or more variables used in the analysis. The analysis was set to Jamovi’s handling of missing data as the default. In addition to reporting descriptive statistics (eg, means, SDs, and frequencies), we conducted inferential statistical analyses to explore relationships and differences among variables.

We applied a paired-sample 2-tailed *t* test to identify differences in AD between ICU admission and postdischarge. Furthermore, correlation analyses were conducted to determine the strength and direction of relationships among transportation difficulties, satisfaction with the frequency of patient updates, mental health indicators (reported AD status), and family interest in mHealth solutions. However, because the data did not meet the assumptions of normality, nonparametric tests were used to assess differences (Wilcoxon *W*) and relationships (Spearman ρ) among variables. Further analysis details are provided in the Results section.

### Ethical Considerations

This study was reviewed and approved by the institutional review board (approval 83549) at the University of Kentucky, Lexington, Kentucky, United States, in accordance with the principles of the Declaration of Helsinki. The participant population was from both within and outside the University of Kentucky. Outside populations were recruited through REDCap advertising networks and through the list of websites that specialize in participant recruitment. Researchers obtained an institutional review board waiver of documentation for the informed consent process. All participation was on a voluntary basis, with no compensation offered. Research presented no more than minimal risk to the participant because it involved only survey and interview questions and obtaining consent would increase the risk related to confidentiality based on the consent document being the only link to any personal health information.

## Results

### Demographics

We used a nonprobability sampling technique, necessitating the collection of demographic information from family member participants. Patients spanned various age groups, with a notable concentration of those aged ≥31 years. The 97 individuals who responded to the survey represented a spectrum of ages, races, ICU patient disease types, and relationships to patients. The data showed a balance of both male and female patients. Race identification among family members was 80% (n=78) White, 18% (n=17) Black, and 2% (n=2) other races.

Regarding medical reasons for patient admission to the ICU, the percentage breakdown of the sample included cancer (n=16, 17%) and COVID-19 (n=13, 13%) as the most frequent, followed by pneumonia or infections (n=9, 9%), myocarditis (n=7, 7%), stroke (n=7, 7%), sepsis (n=7, 7%), accidents or injuries (falls, burns, cuts, or gunshot wounds; n=7, 7%), overdose or substance abuse (n=6, 6%), diabetes (n=3, 3%), meningitis (n=2, 2%), and other conditions (n=20, 21%). The largest group of patient relatives who spent time at the bedside were parents or guardians (n=34, 35%), followed by spouses (n=14, 14%), grandparents (n=12, 12%), and other relatives (n=37, 39%).

### Normality of Data

**Figure 1:**
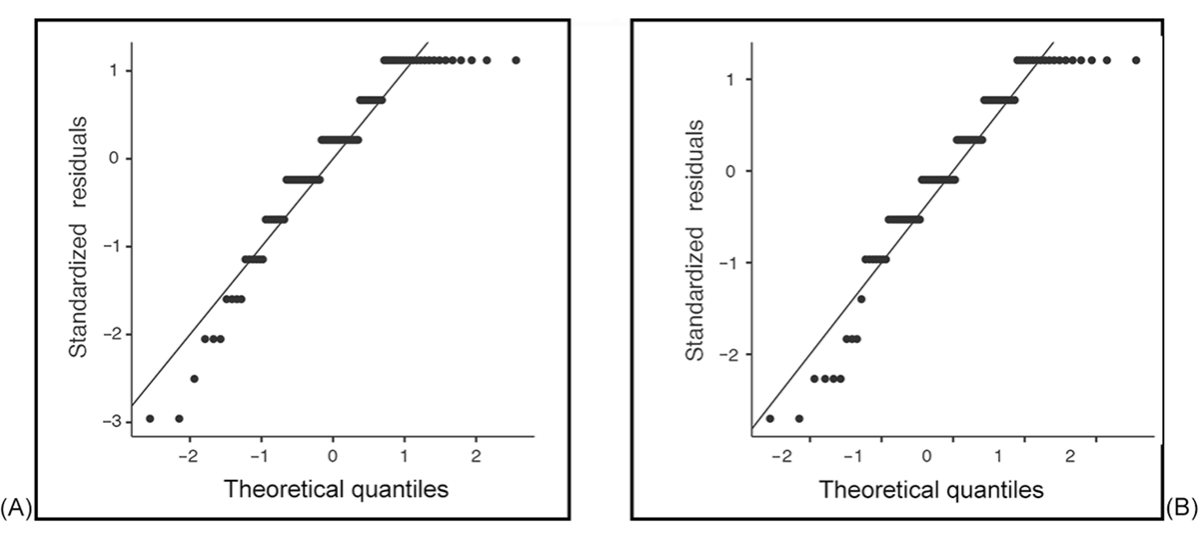
Q-Q plots of anxiety (A) and depression (B) during the patients’ stay.

**Figure 2:**
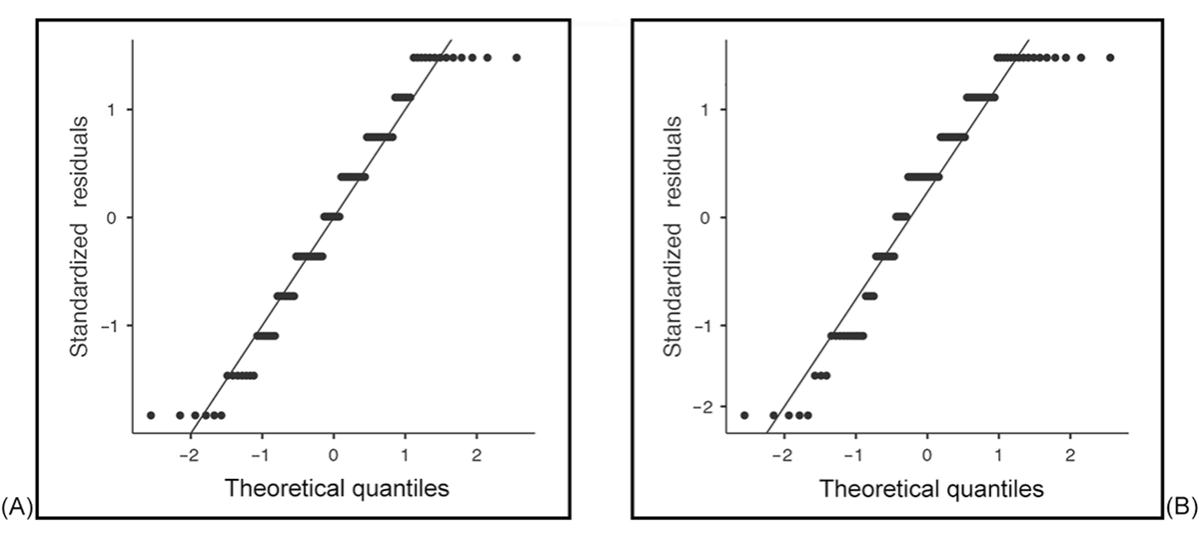
Q-Q plots of satisfaction with the frequency of communication of medical information via telephone (A) and verbally (B) while families are in the intensive care unit.

**Figure 3:**
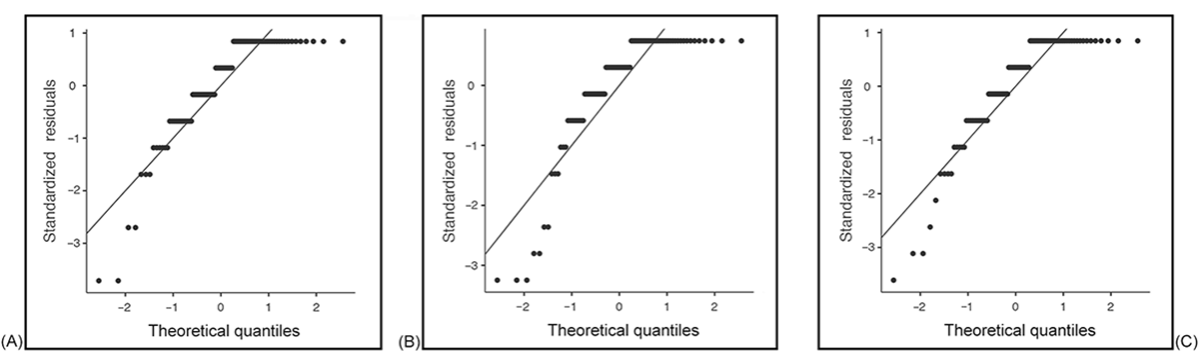
Q-Q plots of general interest in the use of mobile health (mHealth; A), an mHealth videoconferencing app (B), and an mHealth feature that connects family members with a social worker (C).

**Figure 4:**
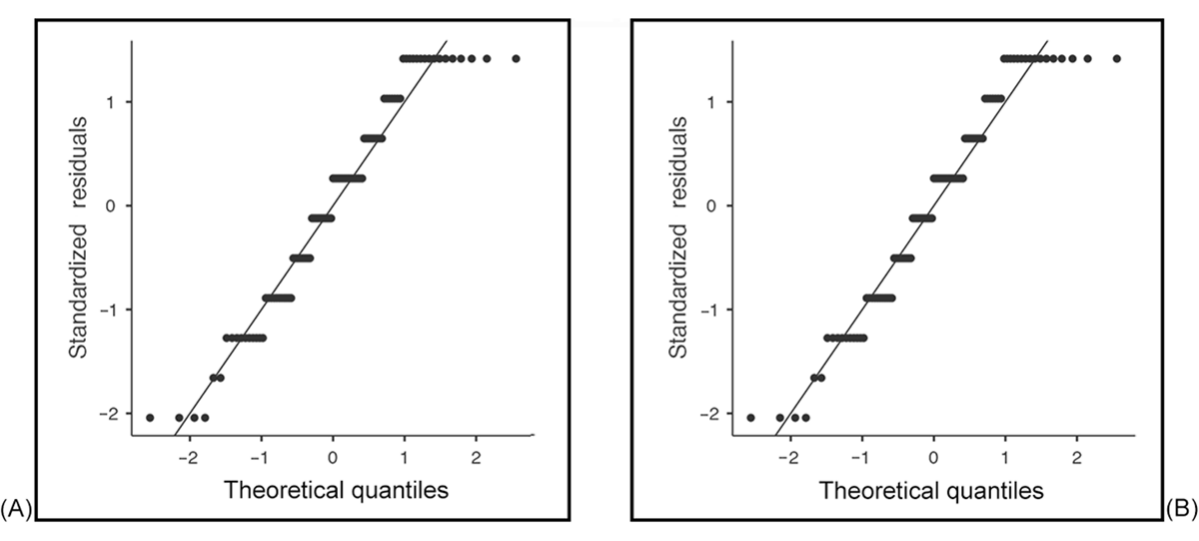
Q-Q plots of anxiety (A) and depression (B) after discharge.

We analyzed the normality of the data by using skewness and kurtosis tests for all dependent variables. Table 1 shows how the data were normally distributed. Values were within the threshold range for both skewness (–1 to +1) and kurtosis (–3 to +3) [54]. We supplemented the skewness and kurtosis statistics with formal normality tests, specifically the Shapiro-Wilk test, which is more appropriate for small to moderate sample sizes. To further support our assessment, we generated *Q*-*Q* plots for the main continuous variables used in parametric analyses. These plots were examined to identify deviations from normality that may not be evident in statistical tests alone. The Shapiro-Wilk test results and *Q*-*Q* plots showed deviation from normality; therefore, nonparametric tests were applied for inferential statistics. Figures 1-4 provide the *Q*-*Q* plots illustrating the data.

**Table 1:** Data normality.

	Descriptive statistics		Skewness (SE)	Kurtosis (SE)	Shapiro-Wilk test	
	Participants (n)	Score (1-10), mean (SD)			Wilcoxon *W*	*P* value
Depression during the patients’ stay in the ICU^a^	95	7.22 (2.30)	−0.84 (0.24)	0.31 (0.49)	0.91	<.001
Anxiety during the patients’ stay in the ICU	95	7.53 (2.21)	−0.93 (0.24)	0.55 (0.49)	0.89	<.001
Depression after the patients’ ICU discharge	95	6.32 (2.60)	−0.18 (0.24)	−0.92 (0.49)	0.94	<.001
Anxiety after the patients’ discharge from the ICU	95	6.68 (2.38)	−0.48 (0.24)	−0.55 (0.49)	0.93	<.001
Satisfaction with the frequency of telephone medical information communication	95	5.98 (2.72)	−0.17 (0.24)	−0.96 (0.49)	0.94	<.001
Satisfaction with the frequency of medical information communication	95	6.62 (2.70)	−0.53 (0.24)	−0.77 (0.49)	0.92	<.001
Overall mHealth^b^ app interest	95	8.34 (1.98)	−1.60 (0.24)	3.07 (0.49)	0.79	<.001
Interest in mHealth videoconferencing app	96	8.32 (2.25)	−1.84 (0.24)	3.07 (0.48)	0.74	<.001
Interest in mHealth app to connect with social workers	96	8.29 (2.02)	−1.50 (0.24)	2.32 (0.48)	0.81	<.001

^a^ICU: intensive care unit.

^b^mHealth: mobile health.

### RQ 1 Results

RQ 1 addresses the degree to which family members were satisfied with available health information and communication provided by ICU clinical staff. The mean score of 2.94 (SD 1.31) on a scale from 1 to 6 shows that, on average, family members received updates once every 7 to 12 hours when away from the hospital (Table 2). Regarding the satisfaction level with the overall frequency of communication with the patients’ family members, we observed a mean score of nearly 7 on average (mean 6.62, SD 2.70) on a scale from 1 to 10. Conversely, phone communication was lower on average. Furthermore, satisfaction with the mental health services provided by the hospital for family members was at a midpoint on a scale from 1 to 10 on average (mean 4.97, SD 2.90). Mental health services as defined in the survey included mental health counseling, social services, and clinical psychologists available to family members on request (Table 2).

**Table 2:** Scores for frequency of patient information communicated and satisfaction with the available patient information communicated, combined with the mental health services provided to the patients’ family members.

Mental health services		Score, mean (SD)
**Frequency of patient information communicated (n=97)** ^a^		
	“How often were you updated by the ICU staff regarding your loved one (family member) health status when you were away from the hospital?”	2.94 (1.31)
**Satisfaction with the available patient information communicated, combined with the mental health services provided to the patients’ family members (n=95)^b^**		
	“How satisfied were you with the frequency of medical information being shared by the ICU nurses or doctors about your family member?”	6.62 (2.70)
	“How satisfied were you with the frequency of phone communication the nurses had with your family while your family member was in the ICU?”	5.98 (2.72)
	“Rate your satisfaction with the mental health services available to your family, when your family member was in the ICU, e.g., mental health counseling, social services, clinical psychologists?”	4.97 (2.90)

^a^Scale: 1=“never,” 2=“once every 1-6 hours,” 3=“once every 7-12 hours,” 4=“once every 13-24 hours,” 5=“once every 25-48 hours,” and 6=“after more than 48 hours.”

^b^Scale: 1=“not satisfied”; 10=“highly satisfied.”

### RQ 2 Results

RQ 2 addresses the level of AD among family members while their loved ones were in the ICU, as well as after discharge. Table 3 shows that the mean scores for AD were elevated (scale from 1-10). Anxiety was consistently higher than depression in both periods (Table 3). Levels of AD were higher among family members during the ICU stay than after discharge. To determine whether this difference was statistically significant, we conducted a paired-sample *t* test using the Wilcoxon *W*. The results confirmed a significant difference between AD levels of family members during the ICU stay and after discharge (Table 4).

**Table 3:** Mental health status of the family members during admission of their loved ones to the intensive care unit and after discharge.

	During admission, mean (SD)^a^	After discharge, mean (SD)^a^
Depression level	7.22 (2.30)	6.32 (2.60)
Anxiety level	7.53 (2.21)	6.68 (2.38)

^a^Scale: 1=low; 10=high.

**Table 4:** Comparison of anxiety and depression levels among family members during the intensive care unit stay and after patient dischargea.

	Wilcoxon *W*	*P* value	Effect size—rank biserial correlation
Anxiety during admission and after discharge	1479^b^	<.001	0.52
Depression during admission and after discharge	1844^c^	.001	0.44

^a^The alternative hypothesis states that the mean of measure 1 differs from the mean of measure 2 (ie, there is a nonzero difference between the 2 measurements).

^b^33 pairs of values were tied.

^c^24 pairs of values were tied.

### RQ 3 Results

Prior research has established that there is difficulty in arranging transportation for family members to ICU clinical sites, which impacts their satisfaction level and frequency of receiving patient information, phone communication, and mental health services [55].

For RQ 3, we examined the correlation between difficulty arranging transportation and satisfaction with the frequency of shared information. Our findings confirmed evidence of a negative relationship between transportation difficulties and satisfaction with the frequency of information provided (*r*=−0.284; *P*=.005). This suggests that, as transportation challenges increase, families become less satisfied with the frequency of patient health information. The lack of frequent visits due to transportation issues may also suggest a heightening of AD and, subsequently, a stronger desire for more frequent updates to compensate for family members’ inability to regularly visit the ICU.

RQ 3b examined the correlation between difficulty in arranging transportation and AD. As performing multiple correlation analyses increases the risk of type I error, the Benjamini-Hochberg procedure was applied to control the false discovery rate at α=.05. As all raw *P* values were identical and very small (*P*=.001), the Benjamini-Hochberg–adjusted *P* values were uniformly .002, which is well below the threshold. Therefore, all tests remained significant after false discovery rate correction, indicating strong evidence against the null hypotheses (Multimedia Appendix 1).

Unlike the correlation observed with satisfaction regarding communication of patient information, we found no significant correlation between transportation difficulties and anxiety (*P*=.15) and depression during the patients’ ICU stay (*P*=.71). However, there was a positive correlation between anxiety levels and depression levels during the patients’ ICU stay (*r*=0.550; *P*=.001). Additionally, AD levels experienced during the ICU stay were significantly and positively correlated with AD levels after patient discharge (Table 5).

**Table 5:** Correlations among family members’ anxiety and depression during patients’ intensive care unit (ICU) stay and after patient discharge.

		Depression during the patients’ stay in the ICU	Anxiety during the patients’ stay in the ICU	Depression after the patients’ discharge from the ICU	Anxiety after the patients’ discharge from the ICU
**Depression during the patients’ stay in the ICU**					
	*r*	1	0.550	0.468	0.417
	*P* value	—^a^	<.001	<.001	<.001
**Anxiety during the patients’ stay in the ICU**					
	*r*	0.550	1	0.528	0.528
	*P* value	<.001	—	<.001	<.001
**Depression after the patients’ discharge from the ICU**					
	*r*	0.468	0.528	1	0.692
	*P* value	<.001	<.001	—	<.001
**Anxiety after the patients’ discharge from the ICU**					
	*r*	0.417	0.528	0.692	1
	*P* value	<.001	<.001	<.001	—

^a^Not applicable.

### RQ 4 Results

Regarding RQ 4, we surveyed family members about their interest in new forms of mobile technology designed to improve information flow and communication from the ICU bedside to the family’s location regarding their loved ones’ health status. Table 6 presents data indicating that family members expressed strong interest in using such a service (mean 8.34, SD 1.98), connecting with bedside nurses via SMS text message and videoconferencing (mean 8.32, SD 2.25), and having easy access to social workers to help manage their AD (mean 8.29, SD 2.03; 1=least interest; 10=high interest).

**Table 6:** Interest in patient information communicated using a new mobile health appa.

	Participants (n)	Score, mean (SD)
“How interested would you be in a mobile app that provides better information and communication for families by sending regular patient health and wellness updates, while their loved one is in a critical care facility?”	95	8.34 (1.98)
“How interested would you be in a communication mobile app that gives families the ability to text or videoconference with the bedside nurse, while their loved one is in the ICU or another in-patient facility?”	96	8.32 (2.25)
“How much would you be interested in a communication mobile app that allows families the ability to connect directly to a social worker or mental health counselor, while their loved one is in the ICU or another in-patient facility?”	96	8.29 (2.02)

^a^Scale: 1=least interest; 10=high interest.

### RQ 5 Results

RQ 5 explored the correlation among satisfaction with communicated patient information, AD experienced by family members, and interest in using an mHealth app. As performing multiple correlation analyses increases the risk of type I error, we applied the Benjamini-Hochberg false discovery rate correction (α=.05) to adjust for multiple comparisons. Adjusted *P* values (*q* values) are reported, and correlations with *q*<0.05 were considered statistically significant. Thus, we found it reliable to proceed with a multiple correlation analysis (Multimedia Appendix 2).

**Figure 5:**
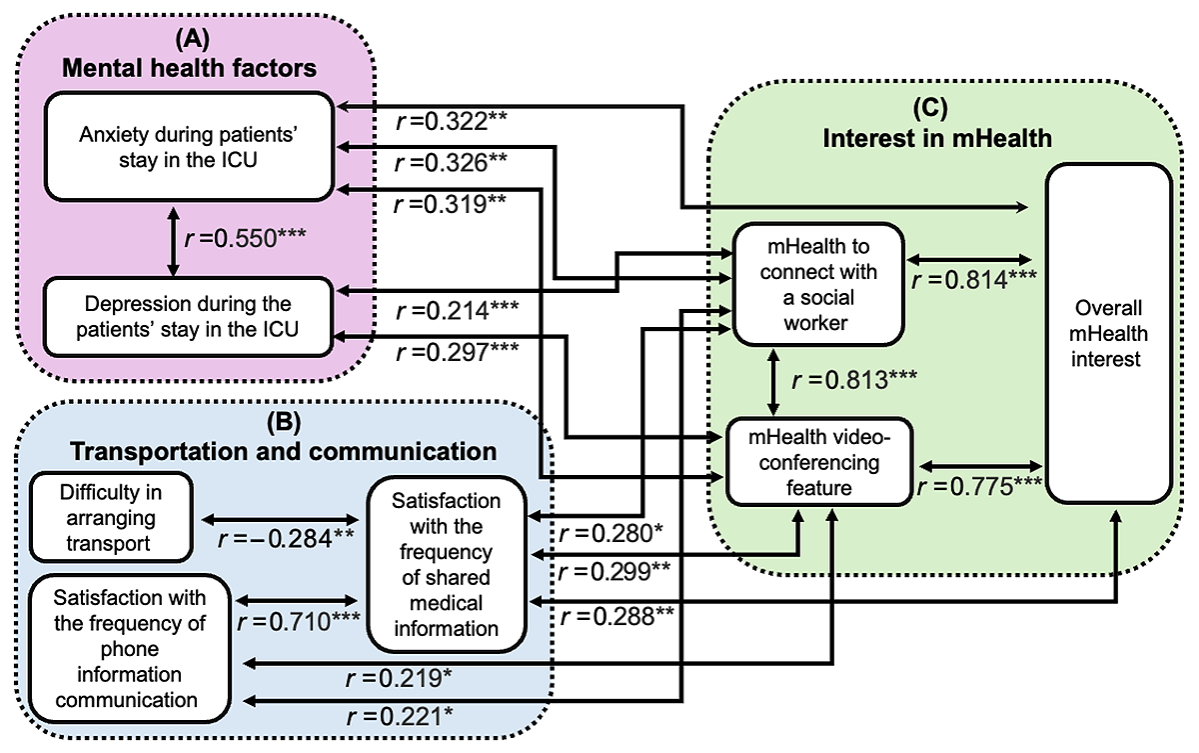
Correlation among 3 variable groups. ICU: intensive care unit; mHealth: mobile health. *P<.05; **P<.01; ***P<.001.

Spearman ρ outcomes revealed significant positive correlations among these variables (*P*<.05; Table 7). We discovered that families experienced higher levels of anxiety during patients’ ICU stays and had significantly greater interest in the use of a mobile app that would provide direct access to social workers (*r*=0.326; *P*<.001), in using an mHealth videoconferencing app (*r*=0.319; *P*=.002), and in overall mHealth app use (*r*=0.322; *P*=.001). We also observed that family members who experienced higher levels of depression during ICU stays had significantly greater interest in SMS text message and videoconferencing communication with the bedside nurse (*r*=0.297; *P*=.003) and an app to connect with social workers (*r*=0.214; *P*=.04). Figure 5 provides a graphic review of the findings.

**Table 7:** Correlation matrix of family members’ anxiety and depression during patients’ intensive care unit (ICU) stay, satisfaction with frequency of medical information communication, and interest in mobile health (mHealth) apps (n=93).

		Anxiety during the patients’ stay in the ICU	Depression during the patients’ stay in the ICU		Interest in app to connect with social workers	Interest in SMS text messaging or videoconferencing app communication	Overall mHealth app interest	Satisfaction with the frequency of telephone medical information communication	Satisfaction with the frequency of medical information communication
**Anxiety during the patients’ stay in the ICU**									
	*r*	1	0.550^a^	0.326^b^		0.319^b^	0.322^b^	0.024	0.064
	*P* value	—	<.001	.001		.002	.001	.82	.54
**Depression during the patients’ stay in the ICU**									
	*r*	0.550^a^	1	0.214^c^		0.297^b^	0.196	−0.058	0.107
	*P* value	<.001	—	.04		.003	.06	.58	.30
**Interest in app to connect with social workers**									
	*r*	0.326^b^	0.214^c^	1		0.813^a^	0.814^a^	0.221^c^	0.280^b^
	*P* value	.001	.04	—		<.001	<.001	.03	.006
**Interest in SMS text messaging or videoconferencing app** **communication**									
	*r*	0.319^b^	0.297^b^	0.813^a^		1	0.775^a^	0.219^c^	0.299^b^
	*P* value	.002	.003	<.001		—	<.001	.03	.003
**Overall mHealth app interest**									
	*r*	0.322^b^	0.196	0.814^a^		0.775^a^	1	0.174	0.288^b^
	*P* value	.001	.06	<.001		<.001	—	.09	.005
**Satisfaction with the frequency of telephone medical information communication**									
	*r*	0.024	−0.058	0.221^c^		0.219^c^	0.174	1	0.710^a^
	*P* value	.82	.578	.03		.03	.09	—	<.001
**Satisfaction with the frequency of medical information communication**									
	*r*	0.064	0.107	0.280^b^		0.299^b^	0.288^b^	0.710^a^	1
	*P* value	.54	.30	.006		.003	.005	<.001	—

^a^*P*<.001.

^b^*P*<.01.

^c^*P*<.05.

Similarly, we observed that satisfaction with phone communication from clinicians was positively correlated with direct access to social workers through mHealth (*r*=0.221; *P*=.03), use of an mHealth videoconferencing app (*r*=0.219; *P*=.03), and satisfaction with the frequency of medical information shared by the staff (*r*=0.710; *P*<.001).

We further confirmed that participants’ interest in an mHealth videoconferencing app was significantly and positively correlated with overall mHealth app interest (*r*=0.775; *P*<.001). Interest in mHealth to connect with social workers was significantly and positively correlated with interest in an mHealth videoconferencing app (*r*=0.813; *P*<.001) and overall mHealth app use (*r*=0.814; *P*=.001). However, there was no correlation between satisfaction with health information communicated and AD.

Finally, our results indicate a positive correlation between satisfaction with the frequency of health information received and satisfaction with phone communication from medical staff (*r*=0.710; *P*<.001). The results further indicate that family members who were more satisfied with the frequency of communication were more interested in using an mHealth videoconferencing app for frequent updates (*r*=0.299; *P*=.003), using an app to connect with social workers (*r*=0.280; *P*=.006), and using an mHealth app for updates about their family members (*r*=0.288; *P*=.005).

## Discussion

### Principal Findings

Our findings confirm that family members experienced high levels of AD both during their loved ones’ ICU stay and after discharge. Notably, AD levels were more pronounced while patients were in the ICU compared to after discharge. This heightened stress was especially evident during the first year of the COVID-19 pandemic, when many ICU patients died without a family member present, leading to increased stress levels among relatives [56]. Although AD can be significant during an ICU stay, the persistence of these issues after discharge, as reported in this study, raises serious concerns. This finding is further confirmed by another recent study reporting that family members exhibited symptoms of posttraumatic stress disorder for up to a year after their loved ones were discharged from the ICU [57].

We emphasize COVID-19 as a primary context for the issues raised due to the recent global impact it had on millions of families. While cancer as a reason for admission to the ICU was noted in 17% (n=16) of cases (COVID-19 constituting n=13, 13% of cases), there are over 200 types of cancer, with the National Cancer Institute identifying breast cancer, lung cancer, prostate cancer, colorectal cancer, bladder cancer, and melanoma as the most common. Due to the noncommunicable nature of these diseases, families do not experience the same types of mental health issues because they are often not isolated from their loved ones. Conversely, COVID-19, within a short period, brought to the fore the severe challenges of family-clinician communication and its impact on the mental health of families.

Our study indicated a maximum mean satisfaction level of only 6.62 (SD 2.70) on a scale from 1 to 10, reflecting a generally low level of satisfaction among family members regarding real-time communication. Difficulties in arranging transportation significantly affected their dissatisfaction with the frequency of updates. This difficulty may be attributed to the challenges of traveling to hospitals from remote areas. This finding aligns with those of previous research that suggests that the psychosocial status of family members can influence their satisfaction with communication.

Satisfaction with existing communication between clinical staff and family members of ICU patients led participants to express interest in a digital solution that could more readily provide real-time bedside health updates, as well as the ability to text and conduct videoconferences. This need was also highlighted during the COVID-19 pandemic, when visitation restrictions underscored the necessity of having alternative means of connecting family members with their loved ones. Virtual visitation via videoconferencing has been proposed as a solution, addressing the challenges faced by family members who struggled with the distance and time constraints associated with in-person visits [56,58].

Additionally, our study found that family members were not satisfied with the adequacy and availability of mental health counseling and social services provided by hospitals. This dissatisfaction further underscores an interest from families in an mHealth solution that facilitates virtual communication with social workers or mental health counseling, particularly for families of critical care patients.

In summary, our findings suggest that families were highly dissatisfied with the frequency of health updates provided by clinical staff, with lower satisfaction reported among those who faced difficulties arranging transportation or lived further from the hospital. Family members experienced high levels of AD during their loved ones’ ICU admission as well as after discharge even though their mental health challenges were reduced.

In this study, modest but statistically significant correlations were observed between family members’ reported mental health status (AD) during the patients’ ICU stay and their interest in using an mHealth app. A similar association was found between participants’ satisfaction with medical communication and their interest in a mobile app. Additionally, difficulty arranging transportation was negatively correlated with family members’ satisfaction with the frequency of medical information sharing. Overall, family members expressed interest in a digital tool that could provide access to real-time bedside information, facilitate communication with bedside nurses, and support connections with social workers.

It is important to note that these findings are based on a nonrandom sample and reflect moderate correlations; therefore, they should be interpreted with caution. Rather than indicating strong evidence of effectiveness, the results suggest potential associations that warrant further investigation. Nonetheless, when considered alongside prior research emphasizing the value of structured interprofessional frameworks in hospital and ICU settings—particularly those using standardized digital tools and empathic communication strategies [59]—the findings highlight directions for future work.

Moreover, prior studies suggest that interventions aimed at mitigating the negative psychological impacts of hospitalization may be beneficial when initiated early during ICU admission. Remote or technology-mediated approaches may improve reach and engagement, whereas individuals with greater needs may benefit from longer or in-person interventions to ensure adequate support and reduce the risk of discontinuation [60].

Building on these insights, we propose that future research explore the development and evaluation of an mHealth intervention designed to support communication between family members and ICU bedside staff. Such an app could offer timely clinical updates and secure messaging or video communication within a HIPAA (Health Insurance Portability and Accountability Act)-compliant platform. Importantly, rather than replicating the limited access often associated with existing mobile electronic medical record services, a family-centered design could acknowledge the central role of family members in patient recovery. However, rigorous studies using randomized designs are needed to assess feasibility, effectiveness, and ethical considerations before such interventions can be recommended for widespread implementation.

### Limitations of the Study

This study has several important limitations that should be considered when interpreting the results. First, our self-selecting sampling strategy may have introduced bias, potentially overrepresenting family members who were more comfortable with technology and, thereby, inflating interest in mHealth apps. Second, the sample size was limited due to the uniqueness of the population studied and the sensitivity of the topic (ie, family members may have found it difficult to fill out the survey due to the need to revisit a prior family event that was psychologically painful). Third, mental health status (AD) was self-reported and was not assessed using standardized scales. Considering these limitations, the findings may be interpreted as exploratory and may not be generalizable to all ICU family populations. Nevertheless, this study offers valuable insights into this important issue and identifies directions for future research.

### Future Research

In parallel with this study, we recently completed a quasi-experimental pilot study that investigated the feasibility, usability, and efficacy of a newly developed mHealth app to reduce the AD of family members of critical care patients with cancer, with a focus on those living in rural Kentucky. This study recruited family members of patients in the Markey Cancer Center, Blood & Marrow Transplant and Cellular Therapy Program, University of Kentucky. As such, our ongoing research includes further mHealth app design, development, and clinical testing of this intervention in both critical care and other long-term health facilities (eg, nursing homes). The aim of this translational research is to provide bedside support to family members living in remote locations throughout Kentucky and Ohio.

## Appendix

Multimedia Appendix 1 *P* values adjusted for multiple comparisons using the Benjamini-Hochberg procedure: correlation between difficulty in arranging transportation and anxiety and depression.

Multimedia Appendix 2 *P* values adjusted for multiple comparisons using the Benjamini-Hochberg procedure: correlation among satisfaction with communicated patient information, anxiety and depression experienced by family members, and interest in using an mHealth app.

## Abbreviations

AD: anxiety and depression
HIPAA: Health Insurance Portability and Accountability Act
ICU: intensive care unit
mHealth: mobile health
REDCap: Research Electronic Data Capture
RQ: research question

## Data Availability

The datasets generated or analyzed during this study are available from the corresponding author on reasonable request.
