# Supplementary material for: Mental Health Needs of Families of Patients in Intensive Care Units and the Role of Mobile Health: Survey Study
Source: JMIR Form Res. 2026 Mar 20;10:e75461. doi: 10.2196/75461 (PMC13049399; doi:10.2196/75461)
Supplement: Multimedia Appendix 1 [file formative_v10i1e75461_app1.docx]

**Appendix A**

P-values Adjusted for Multiple Comparisons using the Benjamini-Hochberg Procedure.

| **Raw P-value** | **Rank (RANK.AVG)** | **BH-adjusted p-value** | **Monotonic BH** | **Significant (FDR 0.05)** |
| --- | --- | --- | --- | --- |
| 0.001 | 3.5 | 0.001714 | 0.001714 | Yes |
| 0.001 | 3.5 | 0.001714 | 0.001714 | Yes |
| 0.001 | 3.5 | 0.001714 | 0.001714 | Yes |
| 0.001 | 3.5 | 0.001714 | 0.001714 | Yes |
| 0.001 | 3.5 | 0.001714 | 0.001714 | Yes |
| 0.001 | 3.5 | 0.001714 | 0.001714 | Yes |
