# Supplementary material for: Mental Health Needs of Families of Patients in Intensive Care Units and the Role of Mobile Health: Survey Study
Source: JMIR Form Res. 2026 Mar 20;10:e75461. doi: 10.2196/75461 (PMC13049399; doi:10.2196/75461)
Supplement: Multimedia Appendix 2 [file formative_v10i1e75461_app2.docx]

**Appendix B**

P-values Adjusted for Multiple Comparisons using the Benjamini-Hochberg Procedure.

| **Raw P-value** | **Rank (RANK.AVG)** | **BH-adjusted p-value** | **Monotonic BH** | **Significant (FDR 0.05)** |
| --- | --- | --- | --- | --- |
| 0.001 | 3.5 | 0.004 | 0.004 | Yes |
| 0.001 | 3.5 | 0.004 | 0.004 | Yes |
| 0.001 | 3.5 | 0.004 | 0.004 | Yes |
| 0.001 | 3.5 | 0.004 | 0.004 | Yes |
| 0.001 | 3.5 | 0.004 | 0.004 | Yes |
| 0.001 | 3.5 | 0.002 | 0.002 | Yes |
| 0.001 | 7 | 0.00329412 | 0.00329412 | Yes |
| 0.002 | 8.5 | 0.00494118 | 0.00494118 | Yes |
| 0.003 | 8.5 | 0.0042 | 0.0042 | Yes |
| 0.003 | 10 | 0.00636364 | 0.00636364 | Yes |
| 0.005 | 11 | 0.007 | 0.007 | Yes |
| 0.006 | 12 | 0.03446154 | 0.03446154 | Yes |
| 0.032 | 13 | 0.033 | 0.033 | Yes |
| 0.033 | 14 | 0.03453333 | 0.03453333 | Yes |
| 0.037 | 15 | 0.037 | 0.037 | Yes |
